# Supplementary material for: Recurrent neural networks enable design of multifunctional synthetic human gut microbiome dynamics
Source: eLife. 2022 Jun 23;11:e73870. doi: 10.7554/eLife.73870 (PMC9225007; doi:10.7554/eLife.73870)
Supplement: Transparent reporting form [file elife-73870-transrepform1.docx]

***eLife’s* transparent reporting form**

We encourage authors to provide detailed information *within their submission* to facilitate the interpretation and replication of experiments. Authors can upload supporting documentation to indicate the use of appropriate reporting guidelines for health-related research (see [EQUATOR Network](http://www.equator-network.org/%20)), life science research (see the [BioSharing Information Resource](https://biosharing.org/)), or the [ARRIVE guidelines](http://www.plosbiology.org/article/info:doi/10.1371/journal.pbio.1000412) for reporting work involving animal research. Where applicable, authors should refer to any relevant reporting standards documents in this form.

If you have any questions, please consult our Journal Policies and/or contact us: [editorial@elifesciences.org](mailto:editorial@elifesciences.org).

**Sample-size estimation**

- You should state whether an appropriate sample size was computed when the study was being designed
- You should state the statistical method of sample size computation and any required assumptions
- If no explicit power analysis was used, you should describe how you decided what sample (replicate) size (number) to use

Please outline where this information can be found within the submission (e.g., sections or figure legends), or explain why this information doesn’t apply to your submission:

We have included the following text in our Methods section describing the choice of sample sizes:

“Sample sizes were chosen based on limitations of experimental throughput as increased number of biological replicates would have reduced the number of possible different communities that could be observed. We chose a minimum of 2 biological replicates (for complex communities in our validation set) and some sample types have up to 7 biological replicates (such as the full community, which was repeated in most experiments as a control for consistency between experimental days).”

**Replicates**

- You should report how often each experiment was performed
- You should include a definition of biological versus technical replication
- The data obtained should be provided and sufficient information should be provided to indicate the number of independent biological and/or technical replicates
- If you encountered any outliers, you should describe how these were handled
- Criteria for exclusion/inclusion of data should be clearly stated
- High-throughput sequence data should be uploaded before submission, with a private link for reviewers provided (these are available from both GEO and ArrayExpress)

Please outline where this information can be found within the submission (e.g., sections or figure legends), or explain why this information doesn’t apply to your submission:

- For genomic DNA extraction, two replicates were used (see line 541, subsection “Genomic DNA Extraction and Sequencing Library Preparation”)
- Other details on the replicates and richness of data are provided in the subsequent discussion
- Inclusion/exclusion of samples: Samples were excluded from further analysis if 0.1% of the reads were assigned to a species not expected to be in the community (indicating contamination) (see line 571, subsection “Bioinformatic Analysis for Quantification of Species Abundance”).
- In summary, the Methods section describes in detail about the experimental setup, including information on replicates, criteria for including/excluding data from further consideration, formation of training and validation sets for machine learning models, etc.

**Statistical reporting**

- Statistical analysis methods should be described and justified
- Raw data should be presented in figures whenever informative to do so (typically when N per group is less than 10)
- For each experiment, you should identify the statistical tests used, exact values of N, definitions of center, methods of multiple test correction, and dispersion and precision measures (e.g., mean, median, SD, SEM, confidence intervals; and, for the major substantive results, a measure of effect size (e.g., Pearson's r, Cohen's d)
- Report exact p-values wherever possible alongside the summary statistics and 95% confidence intervals. These should be reported for all key questions and not only when the p-value is less than 0.05.

Please outline where this information can be found within the submission (e.g., sections or figure legends), or explain why this information doesn’t apply to your submission:

We have included the requisite descriptions in the Figure captions for all experiments depicted in Figures. For some Figures, we included tables of p-values and N in the Supplemental Tables (Table S3), referenced in the corresponding figure caption. We also have included the same for any statistical tests described in the text but not shown in a figure as a parenthetical following the reported results of each test. We have reported exact p-values in all instances unless the p-value was smaller than the numerical precision of the software used (i.e. software returns p=0), where we instead state “p<0.0001”.

(For large datasets, or papers with a very large number of statistical tests, you may upload a single table file with tests, Ns, etc., with reference to sections in the manuscript.)

**Group allocation**

- Indicate how samples were allocated into experimental groups (in the case of clinical studies, please specify allocation to treatment method); if randomization was used, please also state if restricted randomization was applied
- Indicate if masking was used during group allocation, data collection and/or data analysis

Please outline where this information can be found within the submission (e.g., sections or figure legends), or explain why this information doesn’t apply to your submission:

Descriptions of splits of examples into “training” and “validation” sets is discussed in the Results section and summarized in Supplementary Table 1.

**Additional data files (“source data”)**

- We encourage you to upload relevant additional data files, such as numerical data that are represented as a graph in a figure, or as a summary table
- Where provided, these should be in the most useful format, and they can be uploaded as “Source data” files linked to a main figure or table
- Include model definition files including the full list of parameters used
- Include code used for data analysis (e.g., R, MatLab)
- Avoid stating that data files are “available upon request”

Please indicate the figures or tables for which source data files have been provided:

Source data and code for all is available at: <https://gitlab.eecs.umich.edu/mayank.baranwal/Microbiome>

Raw Illumina sequencing data is available at:

<https://zenodo.org/record/5529327>

DOI: 10.5281/zenodo.5529327
